# Antidepressant Potentials of Components from* Trichilia monadelpha *(Thonn.) J.J. de Wilde in Murine Models

**DOI:** 10.1155/2018/6863973

**Published:** 2018-04-22

**Authors:** Kennedy Kwami Edem Kukuia, Jeffrey Amoako Mensah, Patrick Amoateng, Seth Kwabena Amponsah, Benoit Banga N'Guessan, Isaac Julius Asiedu-Gyekye

**Affiliations:** Department of Pharmacology and Toxicology, University of Ghana School of Pharmacy, College of Health Sciences, University of Ghana, Accra, Ghana

## Abstract

*Trichilia monadelpha* is a common medicinal plant used traditionally in treating central nervous system conditions such as epilepsy, depression, pain, and psychosis. In this study, the antidepressant-like effect of crude extracts of the stem bark of* T. monadelpha *was investigated using two classical murine models, forced swimming test (FST) and tail suspension test (TST). The extracts, petroleum ether, ethyl acetate, and hydroethanolic extracts (30–300 mg/kg,* p.o.*), standard drug (imipramine; fluoxetine, 3–30 mg/kg,* p.o.*), and saline (vehicle) were given to mice one hour prior to the acute study. In a separate experiment the components (flavonoids, saponins, alkaloids, tannins, and terpenoids; 30–300 mg/kg,* p.o.*) from the most efficacious extract fraction were screened to ascertain which components possessed the antidepressant effect. All the extracts and components significantly induced a decline in immobility in the FST and TST, indicative of an antidepressant-like activity. The extracts and some components showed increase in swimming and climbing in the FST as well as a significant enhancement in swinging and/or curling scores in the TST, suggesting a possible involvement of monoaminergic and/or opioidergic activity. This study reveals the antidepressant-like potential of the stem bark extracts and components of* T. monadelpha*.

## 1. Introduction

Depression, a serious psychiatric condition characterized by anhedonia, low mood, and feelings of guilt and of worthlessness, is a leading cause of disability worldwide and has a very significant impact on morbidity, mortality, and health care cost [1]. According to the World Health Organization, depression is projected to be the leading cause of disability worldwide by the year 2020 [2].

The pathophysiology of the disease is very complex and not fully understood [2, 3]. Several evidences support the notion that disconcertion in serotonin, noradrenaline, and dopamine neurotransmission is responsible for the observed symptoms of depression. Drugs like tricyclic antidepressants, monoamine oxidase inhibitors, and selective serotonin reuptake inhibitors are widely prescribed for depressed patients; however, the efficacy of most of these medications is limited and it takes a number of weeks for these drugs to produce clinically meaningful improvement in the symptoms [4, 5]. In addition, most of these medications are associated with several side effects including dizziness, nausea, sexual dysfunction, dry mouth, and constipation [6]. The situation is further compounded by the fact that an estimated one-third of patients treated with antidepressant fail to show a relief of symptoms [7]. These factors accentuate the need to search for newer, more efficacious, and better tolerated antidepressants [8].

Plants remain the world's chief source of drugs and their usage in alternative medicine is on the rise [9]. Medicinal plants may provide essential alternative sources for treating depression with greater efficacy and fewer side effects [10, 11]. These medicinal plants produce diverse range of biologically active components that demonstrate beneficial pharmacological effects in man [12]. Several advantages of plant components with regard to the discovery of antidepressants have been reported and the antidepressant activity of plant phytochemical in particular has attracted tremendous attention in recent years [13]. In recent years, phytochemicals such as flavonoids, saponins, alkaloids, and terpenoids extracted from some medicinal plants have been reported to exhibit antidepressant-like effect in various animal models of depression [14–17].

The stem bark of* Trichilia monadelpha *(Thonn.) J.J. de Wilde (family: Meliaceae) is used traditionally in the treatment of some central nervous system (CNS) conditions such as depression, epilepsy, psychosis, pain, and inflammation [18]. Some of the investigated pharmacological effects of the plant include antioxidant, wound healing, analgesic, anti-inflammatory, and antianaphylactic properties [19, 20]. Although oxidative stress, inflammation, and so on have been implicated in depression, the antidepressant potential of* Trichilia monadelpha*, which has shown antioxidant and anti-inflammatory activities, is yet to be investigated. The study thus seeks to investigate the antidepressant activity of the crude extracts and components of the stem bark of* T. monadelpha *using the two classical acute models of depression, the forced swimming, and tail suspension tests.

## 2. Materials and Methods

### 2.1. Preparation of Stem Bark Extracts

The stem bark (together with other plant parts) of* T. monadelpha *was collected from Bomaa, Brong-Ahafo Region, Ghana (7°05′06.60′′N, 2°10′01.66′′W), and authenticated at the Ghana Herbarium, Department of Botany, University of Ghana (Voucher number DPT/JM/001). The plant bark was then chopped into pieces, sun dried for fourteen days, and pulverized into fine powder. The powdered plant bark was serially extracted with petroleum ether at 40–60°C, ethyl acetate, and 70% ethanol over a 24-hour period using the cold maceration technique. The resulting extracts were subsequently concentrated under reduced pressure at 40–60°C to a dark brown syrupy mass in a rotary evaporator. The syrupy mass was further dried using water bath and kept in a desiccator till ready for use. The resulting yields were 0.49% w/w for petroleum ether extract (PEE), 0.6% w/w for ethyl acetate extract (EAE), and 7.3% w/w for hydroethanolic extract (HEE).

### 2.2. Animal Husbandry

ICR mice (20–25 g) were obtained from the Noguchi Memorial Institute for Medical Research, University of Ghana, and kept at the animal house of the School of Biomedical and Allied Health Science, University of Ghana. The animals were kept in stainless steel cages (47 cm × 34 cm × 18 cm) under standard conditions: 12 hr light/darkness cycles, access to normal commercial feed and water ad libitum, and a constant temperature (20–23°C). All animals used in this study were handled according to the Guide for the Care and Use of Laboratory Animals [21]. Ethical clearance was received from the National Institutional Animal Care and Use Committee, Noguchi Memorial Institute for Medical Research, University of Ghana, with protocol number 2014-02-4N.

### 2.3. Preliminary Phytochemical Screening

The extracts were screened for the presence of phytochemical constituents such as alkaloids, saponins, glycosides, tannins, sterols, flavonoids, and terpenoids as described by Trease and Evans [22].

### 2.4. Place and Time of Experimentation

All behavioural studies were done with experimentally naïve mice at the Neuropsychopharmacology Research Laboratory, Department of Pharmacology and Toxicology, University of Ghana School of Pharmacy, and in the light cycle between 7:30 a.m. and 3:30 p.m.

### 2.5. Drugs

Fluoxetine hydrochloride (Prozac) was from Eli Lilly and Co., Basingstoke, England; imipramine hydrochloride (Tofranil) from Mallinckrodt Pharmaceuticals, Ireland.

### 2.6. Primary Observation Test

The behavioural and neuroactive effect of the extracts was first evaluated according to standardized observation grid similar to that described by Irwin [23]. ICR mice were randomly divided into various groups (*n* = 7) and kept in the experimental environment for 7 days to acclimatize. Animals were then fasted overnight but had access to water ad libitum and then treated orally with the extracts at various doses (30–3000 mg/kg). The mice were observed at 0, 15, 30, 60, 120, and 180 min, up to 24 hours after treatment for general changes in behaviour and physiological function as well as mortality. The animals were subsequently assessed for behaviours related to neurotoxicity, CNS stimulation, and depression. Effects on autonomic functions were also noted as well as the lethality of the extracts. From this test the right doses of extracts were selected for the subsequent experiments.

### 2.7. Tail Suspension Test (TST)

The TST was carried out as previously described by Steru et al. [24]. Mice were allowed to acclimatize to the room for 3.5–4 hours before the test. Mice were treated with the various extracts (30–300 mg/kg), fluoxetine (3–30 mg/kg), and vehicle (saline; 10 ml/kg). One hour after oral administration of the test drugs, mice were subsequently suspended be the tail individually from a horizontal bar (distance from floor is 30 cm) using adhesive tape (distance from tip of tail is 1 cm). Duration of immobility (defined as the absence of all movements except those required for respiration), curling, and swinging behaviour was recorded by an observer for 5 min from video recordings of the test with the aid of public domain software JWatcher Version 1.0. Decline in immobility score was the index for antidepressant effect; increase in curling behaviour was suggestive of opioidergic activity.

### 2.8. Forced Swimming Test (FST)

The FST was based on that described by Porsolt et al. [25]. Mice were also divided into groups of 7 animals each and received the vehicle (saline), various extracts (30–300 mg/kg), or reference drug fluoxetine (3–30 mg/kg). One hour after the oral administration of the test drugs, mice were then gently dropped individually into transparent cylindrical polyethylene tanks (25 cm high, 10 cm internal diameter) containing water (20–23°C) up to a level of 20 cm and allowed to swim for 5 min. Each session was then recorded by a video camera suspended approximately 100 cm above the cylinders. The mean immobility score (when the mouse was floating upright and made only small movements to keep its head above the water), swimming score (active horizontal movements), and climbing score (active vertical movements) during the 6 min test were then scored, with the aid of public domain software JWatcher Version 1.0 (University of California, Los Angeles, USA, and Macquarie University, Sydney, Australia. Available at http://www.jwatcher.ucla.edu/). A reduction in immobility score was an indication of antidepressant effect. An increase in climbing score without commensurate change in swimming behaviour is suggestive of adrenergic mechanisms while an increase in swimming score without change in climbing suggests serotoninergic interactions.

### 2.9. Extraction of Components from the Most Efficacious Crude Extract

#### 2.9.1. Saponins (by the Method of Nahapetian and Bassiri [26])

Fifty (50 g) of hydroethanolic extract was dispersed in 500 ml of 20% ethanol. The suspension was then heated over a hot water bath for 4 hours with continuous stirring at about 55°C. The mixture was filtered and the residue reextracted with another 500 ml of 20% ethanol. The combined extracts were then reduced to 40 ml over water bath at about 90°C. The concentrate was then transferred into a 500 ml separating funnel and a volume of 50 ml diethyl ether was added and shaken vigorously. The aqueous layer was recovered while the ether layer discarded. The purification process was repeated. Sixty (60) ml of n-butanol was added to the remaining fraction. The resulting solution was washed twice with 10 ml of 5% aqueous sodium chloride. The remaining solution was heated in a water bath. After evaporation the sample was dried in the oven into a constant weight.

#### 2.9.2. Alkaloids (by the Method of Obadoni and Ochuko [27]; Harborne [28])

Fifty (50 g) of the hydroethanolic extract was weighed into a beaker. A volume of 1000 ml of 10% acetic acid in ethanol was added, covered, and allowed to stand for 4 hours. The solution was filtered and the resulting filtrate was concentrated on a water bath to about one-quarter of the original volume. Concentrated ammonium hydroxide was added drop wise until the formation of a precipitate was complete. The whole solution was allowed to settle and the precipitate was collected and washed with dilute ammonium hydroxide and then filtered. The residue was then collected and dried as alkaloids.

#### 2.9.3. Flavonoids (by the Method of Bohm and Kocipai-Abyazan [29])

Fifty (50 g) of the hydroethanolic extract was twice extracted with 500 ml of 80% aqueous methanol at room temperature. The whole solution was filtered through Whatman filter paper number 42 (125 mm). The filtrate was later transferred into a crucible and evaporated into dryness over a water bath and weighed to a constant weight.

#### 2.9.4. Terpenoids (by the Method of Ferguson [30])

An amount of the hydroethanolic extract, 50 g was soaked in ethanol for 24 hours. The resulting solution was filtered; the filtrate was extracted with petroleum ether. The ether extract was treated as total terpenoids.

#### 2.9.5. Tannins (by Method of Strumeyer and Malin [31])

Fifty (50 g) of crude hydroethanolic extract was weighed into a flask and 500 ml of 80% acetone (v/v) was added. The resulting solution was placed in a water bath at 70°C and carefully shaken for 15 minutes. After cooling, the supernatant was decanted carefully. The extraction was repeated twice and the supernatants were combined and concentrated using a rotary evaporator at 40°C to obtain the crude phenolic fraction. The crude phenolic extract (2.5 g) was dissolved in 20 ml of ethanol and applied on a column packed with 40 g of Sephadex LH-20 gel. Ethanol was used as first eluent, to allow the removal of lower molecular weight phenolic compounds. Then 50% acetone in water (v/v) was used to elute tannins.

### 2.10. Statistical Analysis

GraphPad Prism for windows version 5.03 (GraphPad Software, San Diego, CA, USA) was used for all data and statistical analysis. *P* < 0.05 was considered statistically significant. Differences in means were analyzed by ANOVA followed by post hoc test. Doses for 50% of the maximal effect (ED_50_) for each drug were determined by using an iterative computer least square method, with the following nonlinear regression (three-parameter logistic) equation:

(1) $$\begin{array}{c} Y=\frac{a+\left(b-a\right)}{\left(1+10^{\left(\log{ED}_{50}-X\right)}\right)}, \end{array}$$

where *X* is the logarithm of dose and *Y* is the response. *Y* starts at *a* (the bottom) and goes to *b* (the top) with a sigmoid shape.

## 3. Results

### 3.1. Phytochemical Screening

Preliminary phytochemical screening revealed the presence of alkaloids, flavonoids, glycosides, saponins, sterols, tannins, and terpenoids in HEE. EAE had alkaloids, glycosides, tannins, sterols, and terpenoids while PEE showed the presence of alkaloids, sterols, and terpenoids (Table 1).

### 3.2. Irwin Test

No signs of toxic effects were manifested during the 24 hour observation period for all the three extracts. Extract-treated mice showed signs of sedation at 300–3000 mg/kg. There were signs of analgesic effects as well frequent urination and defecation in all three extracts. No death was also recorded after 24 hours for all the administered doses in all the three extracts (Table 2).

### 3.3. Neurobehavioural Effects of the Crude Extracts in the Tail Suspension Test (TST)

#### 3.3.1. Mean Immobility Score

**Figure 1:**
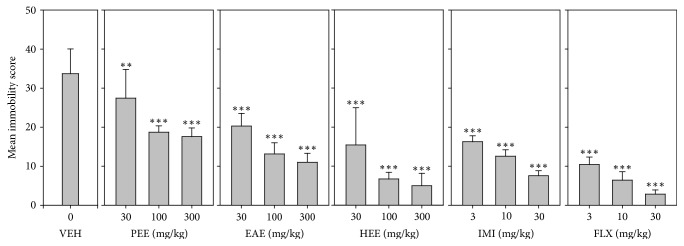
Effects of the extracts, PEE (30–300 mg/kg), EAE (30–300 mg/kg), and HEE (30–300 mg/kg), fluoxetine (3–30 mg/kg), and imipramine (3–30 mg/kg) on immobility score in TST. Data are represented as group means ± SEM (*n* = 7). Significantly different from vehicle: ^*∗∗∗*^*P* < 0.0001; ^*∗∗*^*P* < 0.001 (one-way ANOVA followed by Newman–Keuls test).

All three extracts (PEE, EAE, and HEE) significantly decreased immobility score (*F*_9,60_ = 55.11 *P* < 0.0001; *F*_9,60_ = 66.02 *P* < 0.0001; *F*_9,60_ = 34.83 *P* < 0.0001, resp.; Figure 1). In the TST, the order of antidepressant efficacy calculated from the log-dose response curves was fluoxetine > imipramine > HEE > EAE > PEE (Table 3).

#### 3.3.2. Possible Contribution of Monoaminergic and Opioidergic Mechanisms in Antidepressant Effect: Mean Swinging and Curling Scores

**Figure 2:**
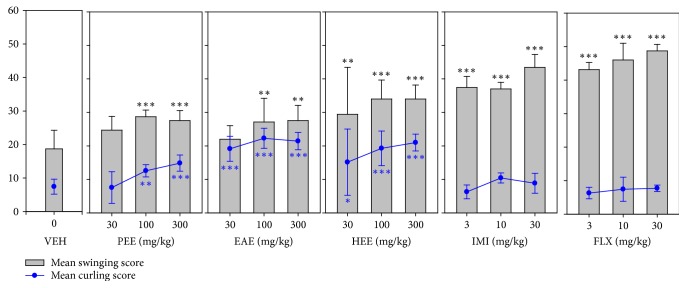
Effects of the extracts, PEE (30–300 mg/kg), EAE (30–300 mg/kg), and HEE (30–300 mg/kg), fluoxetine (3–30 mg/kg), and imipramine (3–30 mg/kg) on mean swinging scores and curling scores in TST. Data are represented as group means ± SEM (*n* = 7). Significantly different from vehicle: ^*∗∗∗*^*P* < 0.0001; ^*∗∗*^*P* < 0.001; and ^*∗*^*P* < 0.05 (one-way ANOVA followed by Newman–Keuls test).

All three extracts (PEE, EAE, and HEE) induced significant increases in swinging (*F*_9,60_ = 57.83 *P* < 0.0001; *F*_9,60_ = 44.30 *P* < 0.0001; *F*_9,60_ = 16.15 *P* < 0.0001, resp.) scores in mice (Figure 2). Similar effects were observed for fluoxetine and imipramine (Figure 2). Again all three extracts exhibited significant increases in curling score (*F*_9,60_ = 8.236 *P* < 0.0001; *F*_9,60_ = 44.85 *P* < 0.0001; *F*_9,60_ = 12.59 *P* < 0.0001, resp.). In contrast, fluoxetine and imipramine did not affect mean curling scores.

### 3.4. Neurobehavioural Effects of the Crude Extracts in the Forced Swimming Test (FST)

#### 3.4.1. Mean Immobility Score

**Figure 3:**
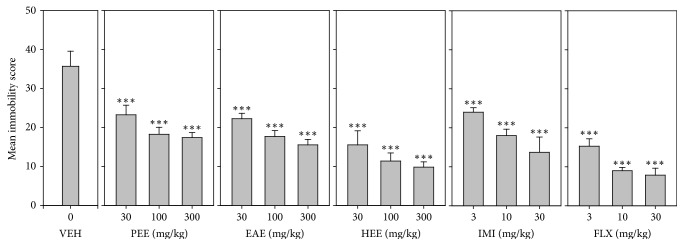
Effects of the extracts, PEE (30–300 mg/kg), EAE (30–300 mg/kg), and HEE (30–300 mg/kg), fluoxetine (3–30 mg/kg), and imipramine (3–30 mg/kg) on immobility score in FST. Data are represented as group means ± SEM (*n* = 7). ^*∗∗∗*^*P* < 0.0001; compared to vehicle-treated group (one-way ANOVA followed by Newman–Keuls test).

All three extracts, (PEE, EAE, and HEE) significantly decreased immobility score (*F*_9,60_ = 87.33 *P* < 0.0001; *F*_9,60_ = 95.33 *P* < 0.0001; *F*_9,60_ = 81.73 *P* < 0.0001, resp.) in a dose-dependent manner (Figure 3).

In the FST, the order of antidepressant efficacy calculated from the dose response curves with regard to immobility was fluoxetine > HEE > imipramine > EAE > PEE (Table 3).

#### 3.4.2. Possible Contribution of Serotoninergic and Noradrenergic Mechanisms in Antidepressant Effect: Mean Swimming and Climbing Scores

**Figure 4:**
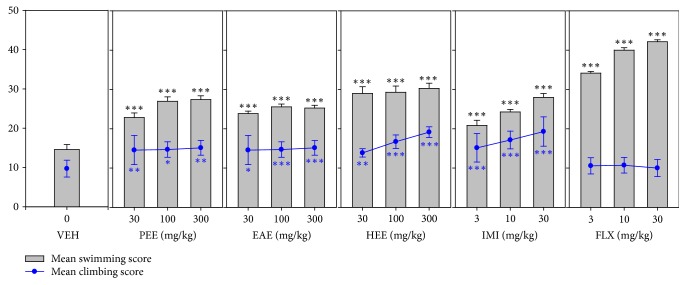
Effects of the extracts, PEE (30–300 mg/kg), EAE (30–300 mg/kg), and HEE (30–300 mg/kg), fluoxetine (3–30 mg/kg), and imipramine (3–30 mg/kg) on mean swimming and climbing scores in FST. Data are represented as group means ± SEM (*n* = 7). ^*∗*^*P* < 0.05; ^*∗∗*^*P* < 0.001; ^*∗∗∗*^*P* < 0.0001; compared to vehicle-treated group (one-way ANOVA followed by Newman–Keuls test).

All three extracts (PEE, EAE, and HEE) showed a significant increase in swimming (*F*_9,60_ = 82.04 *P* < 0.0001; *F*_9,60_ = 108.1 *P* < 0.0001; *F*_9,60_ = 55.06 *P* < 0.0001, resp.) scores. Both fluoxetine and imipramine induced increased swimming behaviour. Similarly, all three extracts significantly increase mean climbing scores just as imipramine. In contrast, fluoxetine did not have an effect on climbing behaviour. Refer to Figure 4.

### 3.5. Neurobehavioural Effects of the Components Extracted from HEE in the Tail Suspension Test (TST)

#### 3.5.1. Mean Immobility Score

**Figure 5:**
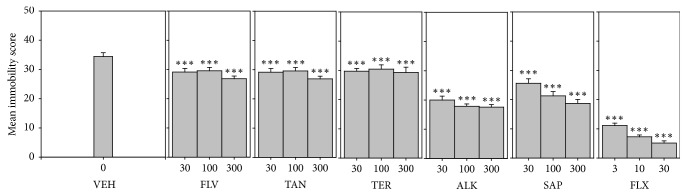
Effects of the components, FLV (30–300 mg/kg), TAN (30–300 mg/kg), ALK (30–300 mg/kg), TER (30–300 mg/kg), and SAP (30–300 mg/kg), and fluoxetine (3–30 mg/kg) on immobility score in TST. Data are represented as group means ± SEM. Significantly different from vehicle: ^*∗∗∗*^*P* < 0.0001 (one-way ANOVA followed by Newman–Keuls test).

Three components, SAP, FLV, and ALK, significantly decreased immobility score (*F*_9,60_ = 82.40 *P* < 0.0001; *F*_9,60_ = 154.2 *P* < 0.0001; *F*_9,60_ = 100.6 *P* < 0.0001, resp.). Refer to Figure 5. In the TST, the order of antidepressant efficacy calculated from the dose response curves with regard to immobility was fluoxetine > ALK > SAP > FLV > TAN > TER (Table 4).

#### 3.5.2. Possible Contribution of Monoaminergic and Opioidergic Mechanisms in Antidepressant Effect: Mean Swinging and Curling Scores

**Figure 6:**
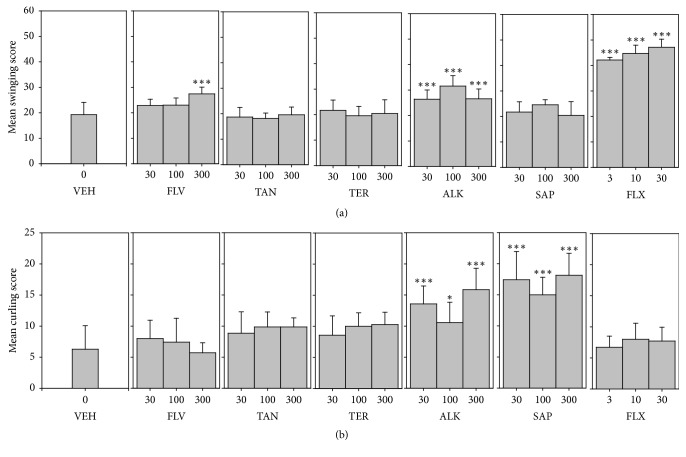
Effects of the components, FLV (30–300 mg/kg), TAN (30–300 mg/kg), ALK (30–300 mg/kg), TER (30–300 mg/kg), and SAP (30–300 mg/kg), and fluoxetine (3–30 mg/kg) on mean swinging scores and curling scores in the TST. Data are represented as group means ± SEM (*n* = 7). Significantly different from vehicle: ^*∗∗∗*^*P* < 0.0001; ^*∗*^*P* < 0.05 (one-way ANOVA followed by Newman–Keuls test).

FLV and ALK showed a significant increase in swinging scores (*F*_9,60_ = 78.70 *P* < 0.0001; *F*_9,60_ = 50.64, resp.) (Figure 6(a)). Similar effect was observed for fluoxetine (Figure 6(a)). In contrast, only SAP and ALK exhibited a significant increase in curling scores (*F*_9,60_ = 17.58 *P* < 0.0001; *F*_9,60_ = 9.655 *P* < 0.0001, resp.) (Figure 6(b)). Fluoxetine did not affect mean curling scores.

### 3.6. Neurobehavioural Effects of the Components Extracted from HEE in the Forced Swimming Test (FST)

#### 3.6.1. Mean Immobility Score

**Figure 7:**
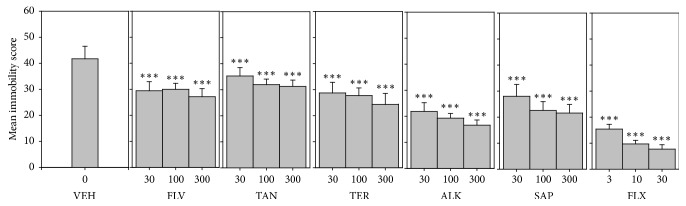
Effects of the components, FLV (30–300 mg/kg), TAN (30–300 mg/kg), ALK (30–300 mg/kg), TER (30–300 mg/kg), and SAP (30–300 mg/kg), and fluoxetine (3–30 mg/kg) on immobility score in FST. Data are represented as group means ± SEM. Significantly different from vehicle: ^*∗∗∗*^*P* < 0.0001 (one-way ANOVA followed by Newman–Keuls test).

All five components (FLV, TAN, ALK, TER, and SAP) significantly decreased immobility score (*F*_9,60_ = 101.7 *P* < 0.0001; *F*_9,60_ = 164.4 *P* < 0.0001; *F*_9,60_ = 91.43 *P* < 0.0001; *F*_9,60_ = 82.12 *P* < 0.0001; *F*_9,60_ = 72.80 *P* < 0.0001, resp.) (Figure 7). In FST, the order of antidepressant efficacy calculated from the dose response curve with regard to immobility score was fluoxetine > ALK > SAP > FLV > TER > TAN (Table 4).

#### 3.6.2. Possible Contribution of Serotoninergic and Noradrenergic Mechanisms in Antidepressant Effect: Mean Swimming and Climbing Scores

**Figure 8:**
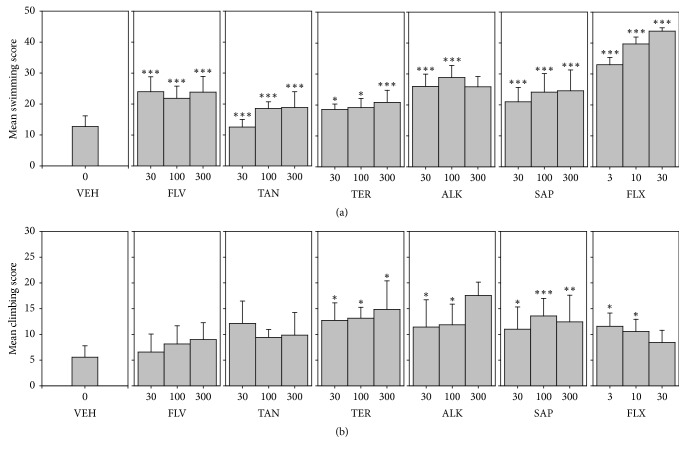
Effects of the components, FLV (30–300 mg/kg), TAN (30–300 mg/kg), ALK (30–300 mg/kg), TER (30–300 mg/kg), and SAP (30–300 mg/kg), fluoxetine (3–30 mg/kg), and imipramine (3–30 mg/kg) on mean swimming and climbing scores in FST. Data are represented as group means ± SEM (*n* = 7). Significantly different from vehicle: ^*∗*^*P* < 0.05; ^*∗∗∗*^*P* < 0.0001; ^*∗∗*^*P* < 0.001 (one-way ANOVA followed by Newman–Keuls test).

All five components (FLV, TAN, ALK, TER, and SAP) induced significant increases in swimming behaviour (*F*_9,60_ = 58.08 *P* < 0.0001; *F*_9,60_ = 93.05 *P* < 0.0001; *F*_9,60_ = 69.09 *P* < 0.0001; *F*_9,60_ = 89.25 *P* < 0.0001; *F*_9,60_ = 42.95 *P* < 0.0001, resp.) as did fluoxetine. Only SAP, TER, and ALK showed a significant increase in mean climbing scores (*F*_9,60_ = 10.52 *P* < 0.0001; *F*_9,60_ = 7.907 *P* < 0.0001; *F*_9,60_ = 12.78 *P* < 0.0001, resp.). See Figure 8.

## 4. Discussion

Results from the present study have demonstrated that oral administration of the hydroethanolic, ethyl acetate, and petroleum ether extracts of the stem bark of* Trichilia monadelpha* possesses significant antidepressant-like activity without demonstrable impairment in locomotor activity or toxic effects.

All three extracts being investigated caused significant reductions in immobility behaviour, which is considered as the principal index for antidepressant efficacy [32] with a concomitant increase in active behaviours such as swimming, climbing, curling, and swinging. Several reports indicate that all antidepressants in clinical use induce a decrease in immobility in rodents while other drugs devoid of antidepressant potential fail to give the same response [33].

The primary aim of the Irwin test, a CNS core battery test, is to assess the effects of a test substance on the behavioural and physiological functions and to reasonably estimate safe dose ranges as well as potential lethal dose of a test drug [23, 34]. In the primary observation test, all three extracts (PEE, EAE, and HEE) demonstrated some sedative effects at higher doses (300–3000 mg/kg) without any deteriorating effect on respiration. This is an indication of a possible CNS depressant effect in all three extracts. Moreover all three extracts also showed analgesic even at lower doses. The observed analgesic effect in the primary observation test confirms earlier reports by Woode et al., [19]. Even at 3000 mg/kg, all three extracts did not induce any observable adverse effects and there were no deaths after 24 hours. These results suggest that the LD_50_ of the three extracts are above 3000 mg/kg, making it safe in the animals used.

In the FST, all three extracts demonstrated antidepressant effect by reducing the duration in immobility and increasing both swimming and climbing scores. Several reports indicate that drugs such as fluoxetine, sertraline, and paroxetine that selectively increase swimming without affecting climbing scores act via the serotoninergic pathway whereas selective noradrenaline uptake inhibitors, desipramine and maprotiline, selectively increased climbing without modifying swimming behaviours of rodents in FST [35]. Thus the observed behavioural effects of all the three extracts seem to suggest that they may be acting via both the serotoninergic and noradrenergic pathway.

In the TST, all three extracts showed antidepressant-like behaviour by inducing a significant reduction in immobility score and also caused increase in both swinging and curling behaviours. Berrocoso and colleagues have reported that compounds that demonstrate increase in curling may be acting via the opioidergic pathway while those that increase swinging score may be enhancing monoaminergic neurotransmission [36]. The findings thus suggest an interplay of monoaminergic and opioidergic activities for all the extracts. Antidepressants which inhibit reuptake of monoamines such as noradrenaline and serotonin have been reported to mediate the enhancement of the opioidergic neurotransmission [37]. The opioidergic system appears to be involved in the antidepressant efficacy of drugs like milnacipran, clomipramine, and so on, whose clinical effectiveness is mediated via *μ* opioid receptors [38]. In addition, several clinical studies have reported on the use of some *μ* opioid receptor agonists as antidepressants in managing refractory depression [39]. The role of the extracts in the enhancement of opioidergic neurotransmission confirms earlier reports that the analgesic effects of these extracts are partially mediated by the opioidergic pathway [19].

Moreover, the roles of inflammation and inflammatory mediators such as tumour necrosis factor (TNF), interleukins (IL), and C-reactive proteins in the pathophysiology of neuropsychiatric conditions like depression have been well documented [40]. Recent scientific study reported that extracts from* T. monadelpha* inhibited TNF-*α* and IL-6 secretion [20]. This may be a plausible mechanism by which the extracts exhibit their antidepressant effect.

Numerous scientific reports have indicated that the pharmacological and therapeutic potentials of medicinal plants are due to the presence of active biological compounds most of which are components [41, 42]. Thus the presence of the phytochemicals in the stem bark of* T. monadelpha* may be responsible for the observed neurobehavioural effects.

The results from the present study indicate that total alkaloid, saponins, flavonoids, terpenoids, and tannins from the most efficacious and potent extract, hydroethanolic extract, possess antidepressant effects in the FST and TST. This suggests that all the components tested contribute to the observed behavioural effect of the hydroethanolic extract. Since the total alkaloid fraction induced increased swimming, climbing, and swinging as well as curling behaviour, it is plausible that its antidepressant effect is dependent on a complex interplay of serotoninergic, catecholaminergic, and opioidergic mechanisms.

It is worth reporting that the efficacy of the individual phytoconstituents was lower than that of the total hydroethanolic extract. It is plausible that the antidepressant efficacy observed in the hydroethanolic extract can be attributed to the synergistic effect produced by the individual active phytoconstituents and possibly via varying mechanisms of action [43].

## 5. Conclusion

The present study shows that components present in the stem bark of* Trichilia monadelpha* have antidepressant-like effect in mice.

## 6. Limitation of Study

This study was conducted in mice. Though there are some genetic similarities between mice and humans, results cannot be extrapolated to humans until further tests are conducted.

## Figures and Tables

**Table 1:** Preliminary phytochemical screening crude HEE, EAE, and PEE from the stem bark of *Trichilia monadelpha.*

Constituents	Inference		
	HEE	EAE	PEE
Alkaloids	Present	Present	Present
Saponins	Present	Absent	Absent
Flavonoids	Present	Absent	Absent
Tannins	Present	Present	Absent
Glycosides	Present	Present	Absent
Terpenoids	Present	Present	Present
Sterols	Present	Present	Present

**Table 2:** Observations in the acute toxicity test after oral administration of PEE. EAE and HEE of *T. monadelpha* in mice.

PEE				EAE				HEE			
Dose (mg/kg)	Mortality D/T	Latency (min)	Observed drug effects	Dose (mg/kg)	Mortality D/T	Latency (min)	Observed drug effects	Dose (mg/kg)	Mortality D/T	Latency (min)	Observed drug effects
0	-	-	-	0	-	-	-	0	-	-	-

30	-	30	Analgesia, urination, defecation	30	-	30	Analgesia, urination, defecation	30	-	30	Analgesia, urination, defecation

100	-	15	Analgesia, urination, defecation	100	-	30	Analgesia, urination, defecation	100	-	30	Analgesia, urination, defecation

300	-	15	Sedation analgesia, urination, defecation	300	-	30	Sedation analgesia, urination, defecation	300	-	30	Sedation, analgesia, urination, defecation

1000	-	15	Sedation, analgesia, urination, defecation	1000	-	15	Sedation, analgesia, urination, defecation	1000	-	15	Sedation, analgesia, urination, defecation

3000	-	15	Sedation, analgesia, urination, defecation	3000	-	15	Sedation, analgesia, urination, defecation	3000	-	30	Sedation, analgesia, urination, defecation

**Table 3:** ED_50_ and *E*_max_ values of extracts and test drugs in TST and FST.

Test	Drug	Decrease in immobility	
		ED_50_	*E*_max_
TST	FLX	1.58 ± 0.2	90.0
	IMI	4.32 ± 1.1	78.01
	PEE	196.10 ± 12.2	42.43
	EAE	66.83 ± 5.6	61.51
	HEE	26.64 ± 3.6	75.44

FST	FLX	2.88 ± 0.6	81.79
	IMI	10.86 ± 1.5	66.74
	PEE	132.20 ± 14.2	54.40
	EAE	108.70 ± 10.6	59.34
	HEE	35.92 ± 2.0	80.55

**Table 4:** ED_50_ and *E*_max_ values of components and test drugs in TST and FST.

Test	Drug	Decrease in immobility	
		ED_50_	*E*_max_
TST	FLX	1.86 ± 0.8	96.0
	IMI	4.58 ± 1.2	95.40
	FLV	912.80 ± 19.2	20.70
	ALK	122.70 ± 11.4	71.10
	SAP	227.20 ± 113.9	55.30

FST	FLX	2.21 ± 1.2	98.30
	IMI	6.80 ± 1.8	98.00
	FLV	376.70 ± 14.2	40.50
	TAN	1348.00 ± 23.7	24.80
	ALK	83.15 ± 6.30	76.40
	TER	408.80 ± 14.8	40.3
	SAP	141.10 ± 12.4	66.1

## Abbreviations

ALK: Alkaloids
EAE: Ethyl acetate extract
FLV: Flavonoids
HEE: Hydroethanolic extract
FLX: Fluoxetine
FST: Forced swimming test
IMI: Imipramine
ICR: Imprinting control region
* p.o.*: Per os
PEE: Petroleum ether extract
SAP: Saponins
TAN: Tannins
TER: Terpenoids
TST: Tail suspension test.
